# Humanitarian Access to Unapproved Interventions in Public Health Emergencies of International Concern

**DOI:** 10.1371/journal.pmed.1001793

**Published:** 2015-02-24

**Authors:** Jerome Amir Singh

**Affiliations:** 1 Centre for the AIDS Programme of Research in South Africa (CAPRISA), University of Kwazulu-Natal, Durban, South Africa; 2 Dalla Lana School of Public Health and Joint Centre for Bioethics, University of Toronto, Toronto, Canada

## Abstract

Jerome Singh considers how regulatory mechanisms can allow access to experimental interventions in humanitarian emergencies such as the Ebola epidemic.

**Summary Points**

- Time-sensitive access to unapproved experimental interventions should be permitted on humanitarian grounds when patients or communities are facing death or irreversible disease progression and no other efficacious diagnostic, preventive, or therapeutic alternative exists.
- Regulatory deficits could stymie time-sensitive efforts to contain public health threats when no efficacious curative, therapeutic, or preventive interventions exist to counter the threat in question.
- United States regulatory mechanisms may provide useful guidance from a regulatory perspective to policy makers grappling with how to adequately prepare for, or respond to, potential or emerging public health emergencies.
- Access to unapproved experimental interventions should be underpinned by a robust monitoring and evaluation component that will inform product development and licensure.
- A global-level rapid-response governance framework for the employment of unapproved interventions in humanitarian contexts should be established as a matter of urgency.

The rampant spread of the Ebola virus in West Africa has prompted the World Health Organization (WHO) to declare the outbreak there a public health emergency of international concern [1]. The United Nation’s Ebola Emergency Response Mission’s (UNMEER) announcement that it would miss its December 2014 deadline to contain the epidemic because of rising numbers of cases [2] illustrates that in the absence of a proven cure, efficacious treatment options or preventive vaccines for stemming Ebola’s spread in Africa and beyond will depend on several factors, including locating, isolating, and caring for those infected with Ebola, tracing their contacts, educating affected communities on safe burial practices, and strict adherence to infection control measures [1,3]. The prioritised, accelerated provision of the unapproved investigational drug Z-Mapp to selected infected individuals and apparent cures in some instances as a result thereof highlight that arresting Ebola’s spread may, by necessity, also have to centre on the accelerated provision of experimental drugs, biological products (including vaccines and biological therapeutics), and devices (including in vitro diagnostics) to affected countries when no adequate, approved, and available alternative to the emergency use of the products in question exists. Such provision must be underpinned by a robust monitoring and evaluation component so that the provision can inform future use and policy on the issue. While this strategy has won WHO endorsement [4], many countries bar the use of unapproved interventions on humans outside of a clinical trial context and lack appropriate regulatory regimes to facilitate the fast-tracked provision of unapproved interventions to those who need them. Also currently lacking at a global level is a coordinating rapid response framework for the employment of unapproved interventions in humanitarian emergencies. In light of this shortcoming, crucial regulatory-related developments in the aftermath of the West African Ebola epidemic are welcomed. These include the US Federal Drug Administration’s (FDA) establishment of an Ebola task force with wide representation from across the FDA to coordinate its Ebola-related activities [5] and a cooperation pledge by members of an interim International Coalition of Medicines Regulatory Authorities in August 2014 [6]. Such initiatives must, however, result in comprehensive, harmonised regulatory mechanisms to ensure that a wide-range of potential Ebola-related interventions are permissible for use in clinical trial and post-trial contexts.

## The Utility of Comprehensive Regulatory Mechanisms to Address Public Health Emergencies

The WHO has urged regulatory authorities to consider the most efficient paths to bring experimental and unapproved products to registration [7]. However, most countries lack regulatory frameworks that permit the use of unapproved interventions on humans outside of systematic research conditions (including in “compassionate use” circumstances) and lack reciprocal recognition agreements with major regulatory agencies, such as the US FDA and the European Medicines Agency (EMA), both of which permit use of such interventions in prescribed circumstances. Such regulatory deficits could stymie time-sensitive efforts to contain public health threats when no efficacious curative, therapeutic, or preventive interventions exist to counter the threat in question. When no other efficacious intervention exists, national authorities globally have an ethical duty to create enabling regulatory frameworks to facilitate the accelerated provision of unapproved investigational interventions in humanitarian contexts to those who may derive benefit therefrom. In settings in which national authorities lack the capacity to devise and enforce such mechanisms, regional structures, such as the African Union (AU), should take an early lead in devising model regulatory templates for countries to adopt. A pledge by African authorities at a meeting of the WHO-led African Vaccine Regulatory Forum (AVAREF) in Pretoria, South Africa, to establish a collaborative mechanism for fast tracking approvals for clinical trials and registration of Ebola-related products in affected countries [8] is thus welcome. The utility of prospectively having such mechanisms in place in the context of public health emergencies is evident in the US, which offers a range of existing regulatory access pathways, including under the country’s medical countermeasure development, preparedness, and response initiative. Some of these regulatory mechanisms are now being postulated and utilised to facilitate time-sensitive access to investigational interventions in Ebola-affected countries. These include (a) the use of unapproved drugs through the Emergency Use Investigational New Drug regulatory pathway and the Emergency Use Authorisation regulatory pathway, (b) the use of approved drugs for unindicated uses, (c) the partial lifting of suspended or halted clinical trials involving investigational drugs, and (d) the approval of new interventions when human efficacy studies are not ethical or feasible. An overview of these mechanisms may provide useful guidance to policy makers grappling with how to adequately prepare for, or respond to, potential or emerging public health emergencies from a regulatory perspective.

### Facilitating Time-Sensitive Access to Unapproved Interventions through the Use of an Emergency Use Investigational New Drug Regulatory Pathway

The US regulatory system permits expanded access to or “compassionate use” of an investigational drug outside of a clinical trial to treat patients with a serious or immediately life-threatening disease or condition that has no comparable or satisfactory alternative treatment option. Access to unapproved interventions under such circumstances may occur through an Emergency Use Investigational New Drug (IND) application or a Treatment Use IND application. In the US, “emergency use” is defined as the use of an investigational drug or biological product with a human subject in a life-threatening situation in which no standard acceptable treatment is available and in which there is not sufficient time to obtain Institutional Research Board (IRB) approval [9]. The Emergency Use IND mechanism allows the FDA to authorize the use of an experimental drug in an emergency situation that does not allow time for submission of an IND application in accordance with standard regulations [10]. It is a mechanism that is also used in instances when patients do not meet the criteria of an existing study protocol or an approved study protocol does not exist. The mechanism exempts the use of the experimental intervention from prior IRB review and approval, provided that such emergency use is reported to the IRB within five working days after the use [11]. This mechanism allows for one emergency use of an experimental intervention at an institution, and any other subsequent use of the investigational product at the institution is subject to prospective IRB review and approval [11]. The use of such a mechanism is invaluable in time-sensitive emergencies such as Ebola outbreaks and was likely the regulatory mechanism utilised to provide the investigational drug Z-Mapp to two Ebola-infected Americans in Liberia [12].

### Facilitating the Time-Sensitive Provision of Investigational Interventions through Emergency Use Authorisations

In the US, the Emergency Use IND mechanism can generally be used in an emergency situation for an individual patient. However, the mechanism is ill-suited for use in mass public health administration—for example, in a universal vaccination campaign against a life-threatening infectious disease taking place in the context of a national emergency or potential national emergency [13]. To manage such instances, the US has established a regulatory mechanism that permits the FDA to approve the emergency use of drugs, devices, and medical products (including diagnostics) that were not previously approved, cleared, or licensed by the FDA or the off-label use of approved products in certain well-defined emergency situations [13]. Such authorisation is issued if no adequate, approved, and available alternative to the emergency use of the product in question exists [14] and if (1) the disease or condition is deemed serious or life-threatening, (2) the totality of scientific evidence reasonably indicates that the product in question may be effective, and (3) the known and potential benefits of the product outweighs its known and potential risks. Such an order was issued for the emergency use of Oseltamivir (Tamiflu), Zanamivir (Relenza), and Peramivir for the treatment and prophylaxis of the 2009 H1N1 influenza virus [14]. Similarly in 2005, an Emergency Use Authorization (EUA) was issued for the emergency use of an unapproved anthrax vaccine for the prevention of inhalation anthrax [15], because it was believed that US military forces faced a heightened risk of attack with anthrax. EUA has also previously been used in the context of potential emergencies for the authorisation of experimental diagnostics for the 2013 coronavirus and the 2013 H7N9 influenza [16]. Such authorisations could be deemed ethically permissible on utilitarian and public health ethics grounds. The value of such a mechanism in the context of Ebola diagnostics was underscored in August 2014. Following an earlier determination in 2006 by the US secretary of homeland security that Ebola virus presents a material threat to the US population sufficient to affect national security [17], on 4 August 2014, the US secretary for health and human services declared that circumstances existed that justified authorizing the emergency use of in vitro diagnostics for the detection of Ebola virus [17]. On the basis of this decision, on 5 August 2014, the US FDA commissioner issued an EUA for the US Department of Defense (DoD) Ebola Zaire Target 1 (EZ1) real-time reverse transcription PCR (RT-PCR) assay for the presumptive detection of Ebola Zaire virus [18].

### Facilitating the Time-Sensitive Provision of Interventions through the Use of Approved Drugs for Unindicated Uses

The off-label use of approved drugs is not equivalent to research or investigational or experimental treatment [19]. Despite the use of approved drugs for unindicated uses being a common and necessary practice in most settings, it is a practice that is not formally regulated, nor formally condoned, in many settings. The US FDA, however, has attempted to clarify the legitimacy of this practice by explicitly condoning the use of approved drugs for unindicated uses in prescribed circumstances. The FDA has noted that “once a [drug] product has been approved for marketing, a physician may prescribe it for uses or in treatment regimens or patient populations that are not included in approved labeling” and that “unapproved” or “unlabeled” uses may be appropriate and rational in certain circumstances [20]. Given this regulatory permissibility, some US scientists have postulated using drugs that have already been approved for the treatment of other diseases for the treatment of Ebola [21]. They argue that unlike experimental Ebola treatments, immunomodulatory drugs have already been approved (thus obviating the need for safety studies), are currently being produced as inexpensive generics, and are available even to countries with basic health care systems. While this proposal has yet to win WHO endorsement, it illustrates the value of having a regulatory regime that permits off-label clinical use outside of an EUA directive. Such an approach may allow for the use of the anti-influenza drug Favipiravir (T-705) as an emergency off-label anti-Ebola agent [22], as is currently the case in France [23], notwithstanding its planned testing as an anti-Ebola agent under clinical trial conditions.

### Facilitating the Time-Sensitive Provision of Investigational Interventions through the Partial Lifting of Suspended or Halted Clinical Trials

The US regulatory system permits the partial lifting of suspended or halted clinical trials [24]. In such instances, the FDA has the discretion to partially lift a trial’s previously declared “hold” in prescribed circumstances. Such a mechanism avoids an “all-or-nothing” approach and permits the clinical trial of an investigational agent to proceed under prescribed conditions, which is invaluable in the context of time-sensitive public health emergencies. The utility of this mechanism is best illustrated in relation to the clinical trial of the anti-Ebola investigational drug TKM-Ebola; the drug was approved for testing in a Phase I clinical trial in 2011 [25], but the trial was then put on hold in early July 2014 to investigate the mechanism behind elevated cytokines in healthy human volunteers receiving the drug [26]. The FDA’s change of status of the drug trial to “partial hold” in August 2014 opened up the possibility for administration to Ebola-infected patients of single ascending doses of the drug, which has demonstrated tolerance in the absence of any steroid-containing premedication at a dose level of 0.3 mg/kg, the maximum tolerated dose in the absence of steroid cover [27].

### Facilitating the Time-Sensitive Use of Investigational Drug Products When Human Efficacy Studies Are Not Ethical or Feasible

The US FDA has the authority to permit the use of drug products in instances when human efficacy studies are not ethical or feasible [28]. In granting such authorisation, the FDA may grant marketing approval for a new drug product for which safety has been established based on adequate and well-controlled animal studies, when the results of those animal studies establish that the drug product is reasonably likely to produce clinical benefit in humans [29]. In assessing the adequacy of animal data, the FDA may take into account other data, including available human data. This mechanism allows the FDA to approve drugs based on efficacy testing in animals and only safety testing in healthy humans. Such a mechanism is particularly valuable in the context of public health emergencies in which following a traditional phased clinical trial evaluative process before a drug can be prescribed for humans is impractical and unfeasible. In 2012 the FDA used its “Animal Rule” mechanism to approve raxibacumab injections for the treatment of inhalational anthrax [30]. This regulatory mechanism has been postulated for use in the context of the Ebola outbreaks [31] and is finding application in respect to the investigational agent BCX4430, which has demonstrated post-exposure efficacy against Ebola virus and Marburg virus disease in rodent models [32] and will soon be the subject of a dose ranging efficacy study in nonhuman primates [33].

## Addressing Governance Gaps

While AVAREC’s pledged collaborative mechanism for fast tracking approvals of clinical trials and the registration of trial products in African countries is welcomed, there is currently no rapid-response governance framework for the employment of unapproved interventions in humanitarian contexts at a global level. Such a system should be created as a matter of urgency. Crucial to such a system will be the development of harmonized data requirements, which will allow for the collection of interpretable data.

However, while time-sensitive access to unapproved experimental interventions should be permitted on humanitarian grounds when patients or communities are facing death or irreversible disease progression and no other efficacious diagnostic, preventive, or therapeutic alternative exists, such access should be underpinned by a robust monitoring and evaluation component that will inform product development and licensure. While the establishment of a governance system on access to unregistered investigational interventions at international, regional, and national levels is crucial to managing public health emergencies of international concern, such a system will be impotent in the absence of competent local regulatory capacity, relevant health system infrastructure, and adequately trained and occupationally protected researchers and health workers at the grassroots level. Uniform import and export regulations as well as civil aviation clearance certification will require particular attention in relation to the transport of biological specimens, especially in countries with less resources, which may lack robust mechanisms. Addressing such gaps in Ebola-affected countries concurrent to intervention provision will entail immersive involvement of international agencies, such as UNMEER and WHO (through initiatives such as AVAREC), donor countries, and nongovernmental organisations, such as Médecins Sans Frontières (MSF), who serve as de facto primary service providers in some humanitarian settings. The eradication of Ebola will necessitate intensive regional cooperation and a well-considered, systematic introduction of experimental interventions to affected countries on the part of the international community.

## Ethics Considerations

Clinical trials on anti-Ebola agents offer trial participants the possibility of accessing unapproved intervention. However, such access raises ethical issues. The introduction of novel interventions in affected countries will require a transparent site selection policy. This will ensure that potential harms and benefits that could arise from the introduction of novel interventions are fairly distributed between affected countries. A transparent site selection policy will facilitate timely prospective community engagement, which is critical in settings where locals distrust authorities and Western medicine. Authorities and investigators will need to ensure that relevant logistics, support infrastructure, and capacity exist in proposed host settings. These factors will be crucial to rollout success and in ensuring the integrity of emerging efficacy data. Investigators must ensure meaningful local scientific collaboration. Investigators and authorities must apply their minds to supply chain logistics, clinical support, and social support in accordance with human rights and ethical norms. The solicitation of informed consent in relation to the use of unproven interventions on individuals should be given particular attention. To this end, investigators should give thought to managing instances when eligible patients lack the ability to provide informed consent autonomously, who should qualify as a surrogate decision-maker when voluntariness may be vitiated by the patient’s dire circumstances, and how to mitigate the occurrence of therapeutic and preventive misconceptions in regards to experimental interventions.

## Conclusions

Time-sensitive access to unapproved experimental interventions should be permitted conditionally, on humanitarian grounds. Regulatory deficits could stymie time-sensitive efforts to contain public health threats when no efficacious curative, therapeutic, or preventive interventions exist to counter the threat in question. A global-level rapid-response governance framework for the employment of unapproved interventions in humanitarian contexts should be established as a matter of urgency. Enacting such measures now will better prepare us to tackle public health emergencies of international concern in the future.

## Abbreviations

AU: African Union
AVAREF: African Vaccine Regulatory Forum
DoD: Department of Defense
EMA: European Medicines Agency
EUA: Emergency Use Authorization
EZ1: Ebola Zaire Target 1
FDA: Federal Drug Administration
IND: Investigational New Drug
IRB: Institutional Research Board
MSF: Médecins Sans Frontières
RT-PCR: reverse transcription PCR
UNMEER: United Nation’s Ebola Emergency Response Mission
US: United States
WHO: World Health Organization
